# The correlation between economic fluctuation, workforce employment and health expenditure in the BRICS countries

**DOI:** 10.3389/fpubh.2022.933728

**Published:** 2022-09-09

**Authors:** Lingyan Gu, Mei-Chih Wang, Fangjhy Li

**Affiliations:** ^1^School of Accounting, Zhejiang Gongshang University, Hangzhou, China; ^2^Department of Insurance and Finance, National Taichung University of Science and Technology, Taichung, Taiwan; ^3^Department of Finance, School of Finance, Hubei University of Economics, Wuhan, China

**Keywords:** BRICS, GDP, economic fluctuation, employment rate, health expenditure

## Abstract

In this paper, we use the Fourier ARDL method (data from 2000 to 2019) to examine whether there is a correlation between economic fluctuation, health expenditure, and employment rate among BRICS countries. Fourier ARDL's model, the same as Bootstrap ARDL model, is to test the long-term cointegration relationship of variables; when there is cointegration, it will test whether there is a causal relationship. When there is no cointegration, short-term Granger causality between variables is tested. Our study shows that, in the long-term, whether South Africa takes economic fluctuation, employment rate or health expenditure as the dependent variable, there is a cointegration relationship with the other two independent variables, but the causal relationship is not significant. In short-term Granger causality tests, the effects of economic fluctuation in Brazil, China, and South Africa on health expenditure lag significantly by one period. Economic fluctuation in Brazil, India and China had a negative effect on employment rate, while South Africa had a positive effect. Health expenditure in Russia and India has a negative effect on employment rate, while China has a positive effect. Employment rates in China and South Africa have a significant positive effect on economic fluctuation, while Russia has a negative effect. India's employment rate has a negative effect on health expenditure, while South Africa's has a positive effect. In short-term causality tests, different countries will exhibit different phenomena. Except for economic fluctuation, where health spending is positive, everything else is negatively correlated, and all of them are positive in South Africa. Finally, we make policy recommendations for the BRICS countries on economic fluctuation, employment rates, and health expenditure.

## Introduction

Due to the particularity of the medical and health service system and its system, it is closely related to social, economic, political, demographic, and cultural and health, as well as comprehensive social management strategies, management paths, and methods. The choice of the health system and different social, economic, political, and demographic development backgrounds should have different health systems, especially the spectrum of disease use, investment approaches, methods, and the number of health resources. There have also been differences in the number of health systems. Health systems also have a tangible impact on health policies and health expenditures. The design of health policies and health systems is also closely related to meeting the needs of residents for health services. The health systems and development models of the “BRICS” health systems are in a period of rapid development due to their socio-economic development (1). From the economic development perspective, each country has gone through a process from a social medical assistance system to a specialized health care system; basically, they have gradually developed to cover some occupational and technical populations. The health system or medical insurance system, from a single medical service or a specific set of medical health service systems, to complex multi-level and multi-level medical service systems, including public health services such as preventive health and health education, health and service content has also been integrated into the medical service system (2).

According to statistics on international health expenditure, in the United States in 1995, medical expenditure accounted for 13.6% of GDP, and in 2017, it increased to about 18% and grew by 32.4%. Similarly, in 1995, medical expenditure accounted for only 6.8% of GDP in Japan, and by 2017, it increased to 11%, growing by 74.6%.^1^ The global pattern of rising real per capita health expenditure appears to be dominated by government sources. Government spending represented about 60% of global spending on health in 2017, up from 56% in 2000. Global public spending on health grew at 4.3% a year between 2000 and 2017. Even so, its growth has been decelerating from 4.9% a year in 2000–2010 to 3.4% in 2010–2017.^2^

**Figure 1:**
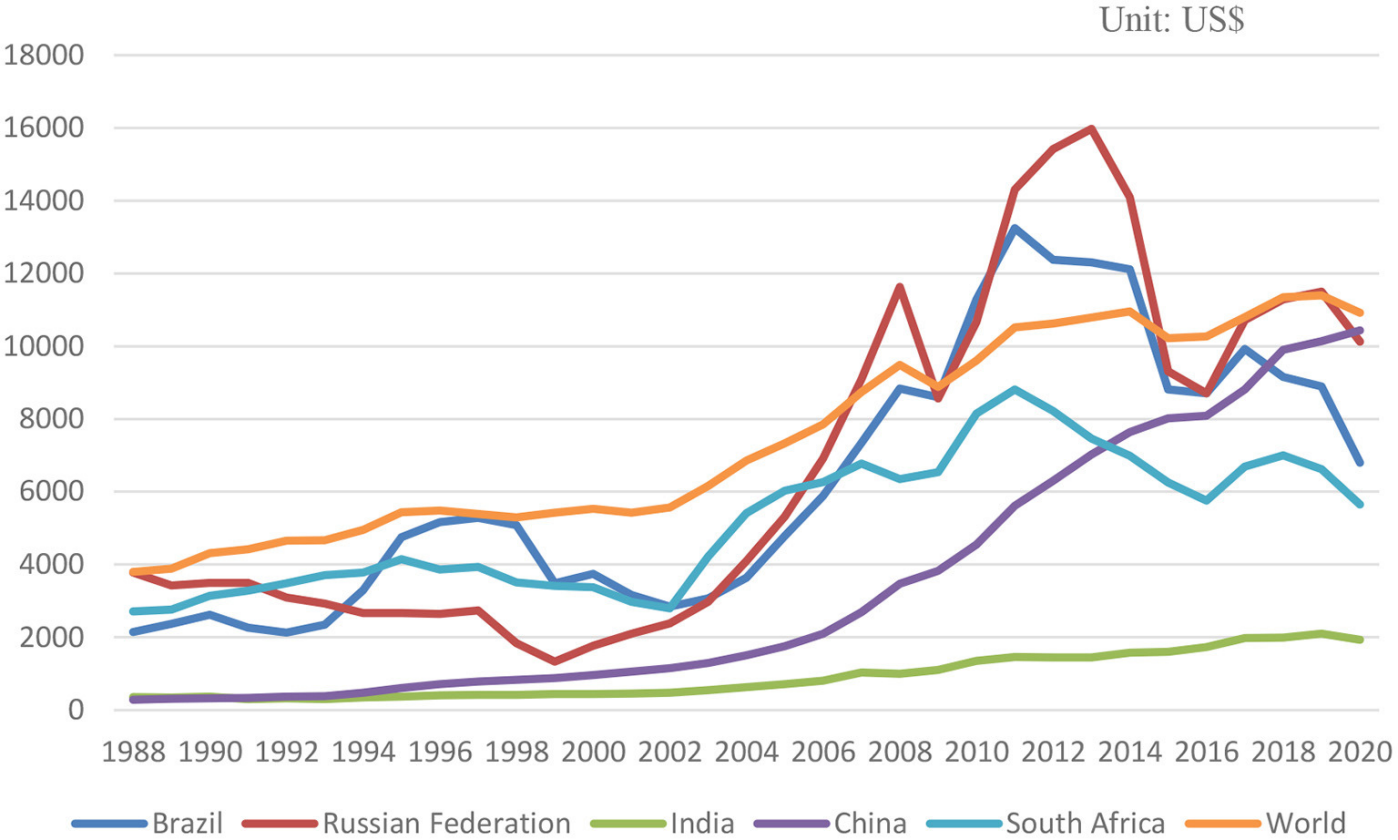
1988-2020 BRICS and world GDP per capita.

Since the end of the 20th century, it has been an international trend to become a market economy country after economic transformation, and the number is considerable. According to International Financial Corporation (IFC)^3^ research, these countries are often referred to as “Emerging Market Countries,” reaching 28 countries, mainly in Asia, Africa, Latin America, the former Soviet Union, and Eastern Europe. The so-called “Emerging Market Countries” are also relative to the “Developed Market Countries,” with about 51 such countries/regions, mainly in Western Europe, North America, East Asia, and Australia. BRICS countries are also “Emerging Market Countries.” The BRICS countries account for 42% of the global population, 23% of GDP, 30% of territories, and 18% of trade. Because the population and a land area of the BRICS countries occupy an important share in the world, it is one of the main driving forces for world economic growth. The BRICS countries far exceed or approach other regional groups of countries in terms of population, economic aggregate and territorial area (see Table 1; Figure 1). At present, the comprehensive economic size of the BRICS countries is larger than that of the Euro area, and will soon be on par with the United States. Over the past few decades, the GDP of the BRICS countries has increased by 179%, and total trade has increased by 94%. From 2008 to 2017, the average annual growth rate of the global economy was 1%, and the average annual growth rate of the BRICS countries was 8%.^4^

**Table 1:** Comparison between BRICS and major regional group.

	**Population**	**GDP**	**Land area**
	**(Billion)**	**(Trillion)**	**(Million sq. km)**
BRICS	3.16	18.34	38.31
NAFTA	0.49	23.48	20.18
EU	0.51	18.77	4.24
ASEAN	0.65	2.95	4.33

Data source: The World Bank, World Bank open data, https://data.worldbank.org/, Date: March 5, 2020.

An effective medical and health system arrangement should not only be beneficial to the resolution of people's physical health and disease risks, but also the continued sustainability of the existing property rights system and economic system. Economic transition refers to the transition of a state of economic operation to a completely different state of economic operation. It refers to a fundamental change in the country's economic structure, economic system, and economic operating mechanism. Economic transformation has changed the old economic system, improved the economic structure, and changed the mode of economic growth. It is a qualitative change in the national economic system and structure. With the reform and transformation of the political and economic systems, the reform of the medical and health systems in the BRICS countries has gradually developed in-depth, and major changes have taken place in deeper areas such as institutional and structural. BRICS countries have a new force in the field of global health (3).

The rest of the paper is organized as follows: Section Literature reviews presents the previous literature, Section Data and methods presents the data and methods, and Section Empirical results and discussion presents the empirical results and discusses some policy implications. Finally, Section Conclusion and policy suggestions summarizes the research.

## Literature reviews

Health expenditure is seen as a variable that constitutes economic growth. Health expenditure is considered an investment theory in human capital and human capital affects economic growth (4). The important factors of magnitude, scholars supporting this growth theory (5), and argue (6) that economic growth is significantly affected by health expenditure (7). Kleiman (8) and Newhouse (9) used cross-sectional data to open up research on the determinants of health expenditure in countries. The data of Newhouse (9) are from 13 countries with similar economic degrees in 1968, 1971, 1971, or 1972, and measured the average gross domestic product per capita. His findings are: (1) More than 90% of the variance in medical expenditure can be explained by the income, so the income is the most important explanatory variable, and the other “non-revenue variables” are relatively unimportant, while in the study by Kleiman (8), the sample the adoption of 16 countries (including poorer countries), the result is also supportive income can explain the growth of most medical expenditures (Kleiman research results are also more than 90% of the variation can be explained by income), other “non-revenue variables” it is not important. (2) The elasticity of health care is between 1.26 (excluding Greece) and 1.31 (including Greece), so health care is a luxury, and the result is the same as Kleiman (8) (gets a health care income of 1.22), this one, as a result, the more affluent countries, the higher the proportion of gross domestic product spent on health care spending. In the past few decades, although many scholars [such as (10–13)] have done more in-depth research on Newhouse (9) research methods and data definitions. The discussions, but the basic conclusions, have not gone beyond the two important findings mentioned above. Health expenditure is a factor that shows the quality of people's life. Since health is an essential issue in people's lives, any expenditure that helps the health of people has a positive impact on welfare (14).

Ezziane (15) studies the comparison of drug expenditures and total health expenditure among BRICS countries, using developed country technology transfers and know-how to locally produce drugs/vaccines and biotechnology entrepreneurship cooperation in the BRICS countries. His research concluded with recommendations to support greater cooperation between BRICS countries and many developing countries to reduce the cost of drug production. In the BRICS countries, health insurance coverage and services covered by health insurance plans are expanding. The overall purchasing power of the BRICS countries has increased, followed by an increase in the affordability of most medical products and services that ordinary citizens usually pay out of pocket. When considering changes in the global health care landscape, slow, and stable economic growth in most mature, saturated markets should also be considered. Multinational health care companies should focus on emerging markets, especially BRICS countries. Investments in emerging markets will remain key to the long-term profits and sustainability of global pharmaceutical companies and medical device manufacturers (16). According to Jakovljevic (17), the rise of the middle class is increasing demand for medicines and new medical technologies, especially in developed urban core areas. The long neglect of the rural population, many of whom live near the poverty line, has ultimately led to more decisive policies to address these issues. BRICS countries' joint health care has successfully increased six times in less than two decades (1995-2012). Local governments in BRICS countries have successfully used social welfare to improve access to and quality of medical services through different mechanisms. The rise of the middle class is increasing demand for medicines and new medical technologies, especially in developed urban core areas. Health insurance coverage has seen major improvements for the first time in these regions. Although the medical affordability of ordinary citizens is expanding, it is not enough to follow the disproportionately rapid growth of subsequent out-of-pocket expenses. This means some serious setbacks affecting the access of poor to health care (16, 18). Tan et al. (19) studied the implementation mechanism of payment reform for medical service providers in developing countries in Asia. This mechanism is used to transform incentives within the health system, which is cost-effectively plagued by inefficient distribution and high out-of-pocket costs of basic services. Their research found that policy design, policy capabilities, local government willingness to adopt policies, and provider autonomy of medical service provider payment reform implementation mechanisms are key background factors that may trigger different policy mechanisms, leading to expected theoretical results or bad incentives.

### Health expenditures and economic fluctuation

**Figure 2:**
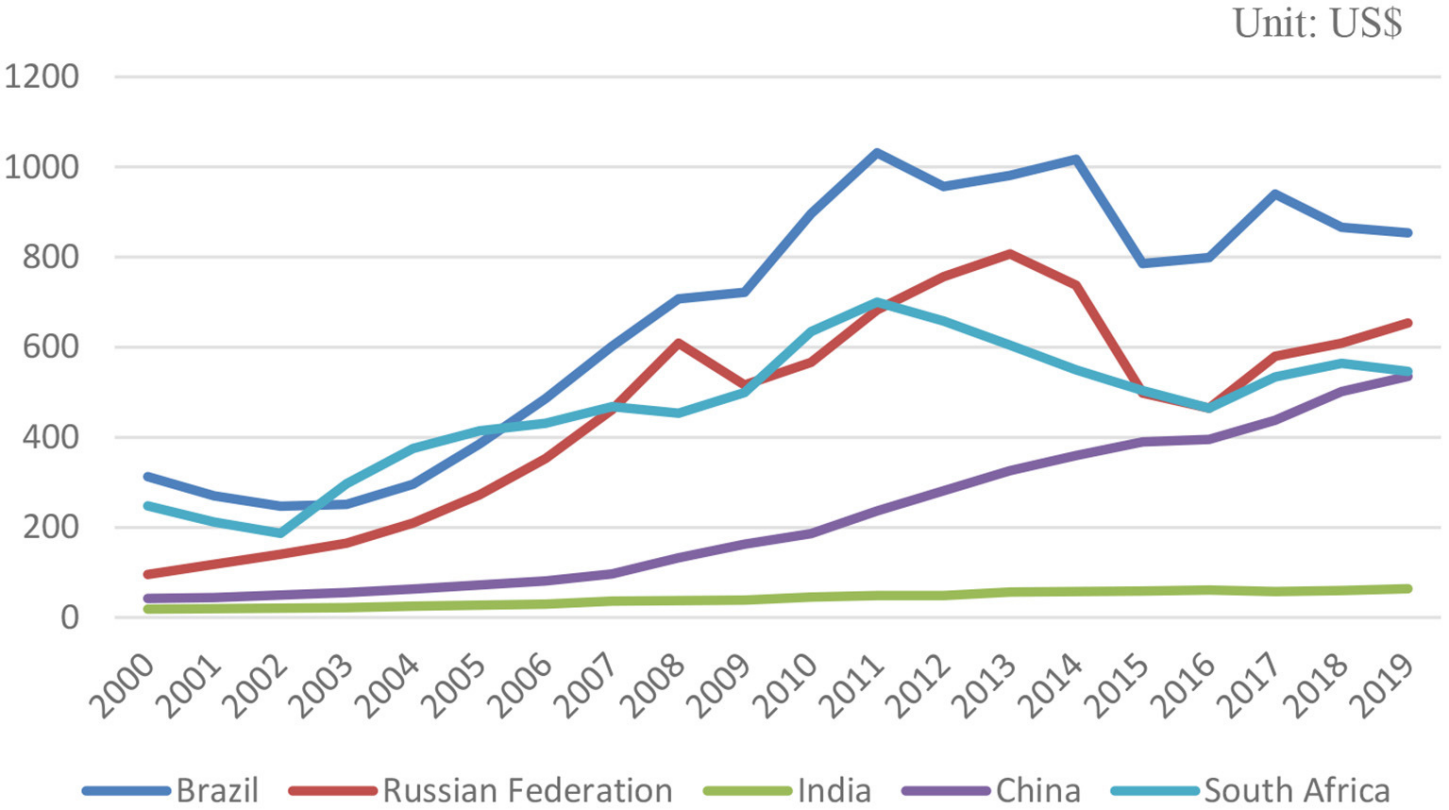
2000-2019 BRICS health expenditure per capita.

Many studies have shown that the proportion of the health expenditure of a country in its GDP is directly proportional to its level of per capita GDP; therefore, the level of economic development in a country has reached the level of expansion of the health services of a country, it will increase with it. In countries with national welfare systems, health costs are relatively low; countries with social insurance are somewhere in between, and market intervention has the highest medical costs. Because in these countries, the expansion of the health sector must obtain government budgets in other regions every year, the government can exercise effective control over the size of the budget. The Inter-American Development Bank conducted a comparative study of the health systems of Latin American and Caribbean countries in the mid-1990s and found that the health status of the people in each country has nothing to do with the implementation of the health system in each country. Blomqvist and Carter's (20) research on whether medical expenditures are luxury goods argues that the demand for health services is regarded as a derivative demand and the basic commodity that consumers value is “good hygiene,” not health services. The level of health input is closely related; the level of total investment is closely related to the level of economic development (2). The rise of new institutional economics is closely related to the existence of many countries with economies in transition and their demand for institutional economics. The essence of the transition is an institutional change or institutional innovation. The rural medical and health system itself is subject to the arrangements and changes of the entire rural ownership and economic system. The growth of the economy symbolizes the increase of national wealth and the improvement of people's wellbeing. From the highest administrative authorities of each country, the annual economic growth of a country is visible to congress. Per capita expenditure continues to rise, and the rate of growth in health expenditure in various countries has long been much higher than the rate of economic growth so that the proportion of health care expenditure to GDP continues to increase. The growth trend of advanced countries in Europe and America is particularly obvious (21). Figure 2 shows the per capita health expenditure of the BRICS countries in US dollars from 2000 to 2019. From the figure, we can see: Brazil declined from 2000 to 2003, began to grow significantly after 2003, and declined in 2011 due to the effect of the subprime mortgage crisis; in 2014, it experienced a relatively large decline, and the overall Brazil is also the country with the highest per capita health expenditure in the BRICS countries. Health expenditure per capita in Russia peaked at $807.07 in 2013 and has since fallen. India's health expenditure per capita has maintained steady but very low growth since 2000, and was only at the level of $63.75 in 2019. China's health expenditure per capita has been growing steadily. Since 2008, there has been a relatively large change in growth. This may be related to China's hosting of the Olympics and its accession to the WTO. China's GDP has grown significantly since then. South Africa, like Brazil, saw a slight rise in health expenditure per capita from 2011 to 2014, but it has not recovered to the 2011 level as of 2019 data.

As a result, health expenditure is seen as an important aspect of providing information on national development. Because of this, the goal of every country in the world is to increase health expenditure (22, 23). Health expenditure promote economic development and the importance of public health expenditures to national economic development (24–27). When the health of people improves, they can increase productivity (18). High-growth countries have increased health expenditure (28, 29). Some countries provide incentives to attract foreign investors. Therefore, economic development is achieved by increasing investment and reducing unemployment (30).

Leu (10) first questioned the argument that Newhouse considered “non-revenue variables” to be unimportant. Leu (10) used the OECD's data from 19 countries in 1974. In addition to the variables obtained, it also added “non-revenue variables,” such as government medical expenditure financing ratio, government supply, urbanization, and proportion of the old and young population. The results show that the yield is still the most important explanatory variable, and the resulting elasticity is between 1.18 and 1.36. Other variables have a significant impact, but the impact is not significant. For example, in the proportion of government financing, when the government departments spend 10% of the total expenditure on medical expenses, the average medical expenditure per person will increase by 2–3%. Other situations are the same. Murthy and Ukpolo (31) used time-series analysis of data from 1960 to 1987 in the United States. First, Dickey and Fuller (32) and Augmented Dickey-Fuller (32) methods were used for single-checking. The unit root phenomenon, if the variable has a unit root phenomenon, represents a time series in which the variable is unstable. After the cointegration analysis, the Maximum Eigenvalue Test and the Trace Test show that there is a cointegration vector (long-term relationship) between the medical expenditure variables and these variables, and the resulting elasticity is 0.77, so the medical health care is a necessity, but GDP variables are not very significant. The aging population and the number of physicians per 100,000 populations are quite significant. There is a positive relationship between population aging and the growth of health expenditure. The number of doctors is negatively related to the growth of health expenditure. The possible reason is that US healthcare lacks price elasticity and supply inducement. Demand does not affect, so increasing the supply of physicians will significantly reduce the market price of health care, which in turn will lead to a decline in health expenditure.

The growth of health expenditure is driven by the following basic questions: birth rate, per capita income, inflation, and so-called “excessive growth,” which is mainly due to advances in medical technology or increased demand for services from patients. “Excessive growth” is responsible for increasing the share of health care in the gross national product, thereby challenging fiscal sustainability. The trend of excessive growth can be seen in health insurance premiums, which continue to grow faster than reductions or wages (33, 34). In recent decades, global economic growth has been driven primarily by the developing world economy. The fastest-growing markets are the top “Emerging Markets” led by the BRICS and N-11 (Bangladesh, Egypt, Indonesia, Iran, South Korea, Mexico, Nigeria, Pakistan, the Philippines, Turkey, and Vietnam) countries (35). Investment in health care is becoming a high priority for global public health. Now, most of the world's real GDP growth and growth in health care expenditure is happening in emerging Eurasia countries that transcend EU borders and extend to the Far East. The increase in medical spending is largely driven by the shared health of mature and emerging economies (36). Yang (37) point out “there may be a non-linear relationship between health expenditure, economic growth and that this relationship may be affected by human capital.” The human capital includes some countries provide incentives to attract foreign investors.

Hansen and King (38) used the time series analysis method to analyze the data of 20 OECD countries from 1960 to 1987, and the explanatory variables were the average per capita health expenditure. The empirical results show that about two-thirds of the data cannot be rejected at the level, and no country's data can completely reject the unit root at the level. In terms of cointegration analysis, there is no long-term relationship between health expenditures and GDP and “non-revenue variables” or between health expenditures and GDP, so the impact of income on health expenditure is denied. Besides, Blomqvist and Carter (20) also found that health expenditure and GDP are a series of unstable. McCoskey and Selden (7) and Gerdtham and Löthgren (13) acknowledged this shortcoming, and early scholars also believed that structural changes are very important because ignoring structural change factors will lead to erroneous unstable timing results, so Jewell et al. (39) used the Panel LM unit root test methodology to determine whether data constancy will be affected when structural changes exist. Also, in terms of GDP, 11 countries have significantly rejected unit-roots, and structural changes occurred in 7 countries, 3 of which also contain two structural changes (Australia, Belgium, Germany), and structural changes. When considering structural changes, the general medical expenditure and GDP data are stable, so the test results of constancy are different from those without considering structural changes, and Jewell et al. (39) believes that after considering the structural changes, the model of medical expenditure and GDP made by the researchers will have more meaningful results.

The financial sustainability challenges facing the health systems of today are still largely due to aging populations, prosperous diseases, mass migrations, rapid urbanization, and medical technology innovation. Although developing countries have good momentum and the “Emerging Markets” of the BRICS countries are leading the way, increasing out-of-pocket costs continue to threaten the affordability of medical services. Even for some of the most advanced OECD countries, expanding universal health coverage can still be a serious challenge. In terms of population health and overall social productivity, health care capacity building has a high but long-term return on investment. Knowledge of health economics promotes smarter and more equitable resource allocation. Contemporary health policy elites believe that achieving universal health coverage remains the primary goal. These conditions have created a strong long-term drive for the inevitable further development of health economics (40). Zaidi and Saidi (41) study the relationship between health expenditure, CO_2_ emissions, and economic growth in sub-Saharan African countries using annual data for the period 1990-2015 and ARDL estimates. The results show that for every 1% increase in per capita GDP, health expenditure increases by 0.332%, while a 1% increase in CO_2_ emissions reduces health expenditure by 0.066 and 0.577%, respectively. There is a bidirectional causal relationship between GDP per capita and between health expenditure and CO_2_ emissions. The results of Granger's causality show that there is a one-way relationship from health expenditure to GDP per capita. Wang and Tao (42) used the data envelopment analysis (DEA) method to measure the static comprehensive efficiency of local government health expenditure in various regions of China from 2007 to 2016. The results show that the ratio of local government health expenditure to fiscal expenditure, per capita GDP and population density have a positive effect on the efficiency of local government health expenditure. Haseeb et al. (43) used ARDL methods and data for ASEAN countries from 2009 to 2018 to analyze the short- and long-term effects of economic growth, environmental pollution, and energy consumption on health and R&D expenditure. The findings show that environmental pollution and economic growth significantly affect R&D expenditure in the short term; any independent variable, namely energy consumption, economic growth, and environmental pollution, has no significant effect on health expenditure in the short term. Wang et al. (44) applied the bootstrapped autoregressive distributed lag (ARDL) cointegration model developed by McNown et al. (45), using annual time-series data for the period 1975-2017. Examines whether there is a long-term relationship between health expenditure, CO_2_ emissions, and gross domestic product (GDP) per capita in 18 Organization for Economic Cooperation and Development (OECD) countries. The main results support bidirectional causality between CO_2_ emissions and GDP growth in the US, Canada, and Germany, between health expenditure and GDP growth in the US and Germany, and between health expenditure and CO_2_ emissions in New Zealand Bidirectional causality. Lee and Kim (46) studied from the perspective of the health law that there must be a performance-based health system in achieving health-related sustainable development goals. One way to strengthen health systems is to improve health governance by expanding the coverage of public health laws. Their findings are that issues with high public health law coverage are health care organizations, infectious diseases, and substance abuse, while low coverage is human reproduction, family health, and oral health. Rechel et al. (2) used panel data and unit root, cointegration and causality method of 161 countries from 1995 to 2014 to study the relationship between GDP and health expenditure The results showed that health expenditure and GDP at different income levels The growth has different causal relationships. Compared with the market, the government has a greater obligation to provide basic health care services. GDP growth will not lead to increased health spending in low-income countries.

### Workforce employment and health expenditure

Margerison-Zilko et al. (47) study the relationship between economic recession and physical and mental health in developed countries (2007-2009). Studies have found that economic recession is harmful to health, especially mental health. Despite the decline in traffic death and population level, macro employment and economic after effects, as well as fertility and self -support, psychological distress and suicide health and incidence. The health of men and ethnic minorities has a greater impact. When the health of people improves, they can increase productivity (18). High-growth countries have increased health expenditure (28, 29). Hone et al. (48) used levels and mortality in Brazilian adults (≥15 years) between 2012 and 2017, and Hodrick-Prescott filters de-trended mortality and unemployment to assess cyclical changes and Control underlying trends. The results showed that the urban adult mortality rate increased from 143.1 deaths per 100,000 in 2012 to 154.5 per 100,000 in 2017, an increase of 8.0%. A 1 percentage point increase in unemployment was associated with a 0.50 increase in all-cause mortality per 100,000 people, mainly due to cardiovascular disease and cancer. Higher unemployment leads to higher death rates. Recession-related increases in mortality were not significant in cities with high expenditure on health and social protection programs. Bíroóa and Elek (49) use Hungarian data to analyze the effects of unemployment on disability insurance, and the impact on health expenditure. The results showed that after the unemployed person occupied a slight decrease in the labor ratio, the outpatient clinic, hospitalization and drug expenditure increased by three times. The medium period of health expenditure increases 20-25 % of the additional disability payment. Drug data shows that the physical health impact, the diagnosis of chronic physical condition (such as hypertension or diabetes), and the deterioration of psychological health can help the surge in health expenditure. Raghupathi and Raghupathi (50) discussed the connection between US public health expenditures and economic performance. Health expenditure can provide better health opportunities, thereby enhancing human capital and increasing productivity, which helps economic performance. The results showed that there was positive correlation between health expenditure and income, and the economic indicators of GDP and labor productivity.

### Economic fluctuation and workforce employment

The labor force is an important variable that affects the potential growth rate of the economy. “The total amount of GDP = labor productivity × labor force.” The growth rate of GDP is affected by the growth rate of labor productivity and labor force. The working-age population and the employment participation rate are important indicators to reflect changes in the labor force. The employment participation rate it will also affect the potential of economic growth. The so-called employment participation rate refers to what proportion of the working-age population is involved in employment. Under the same model conditions of the above-mentioned economies, the analysis result of the employment participation rate is that the high-speed economy the growth has also been accompanied by an increase in the employment participation rate. Evans et al. (51) studied the relationship between the labor force participation rate and GDP in Australia, and understanding the nature of this cyclical relationship between participation and economic activity is important for determining the extent of labor market slack and predicting how the economy will respond to changes in economic conditions. When economic conditions improve, more people enter the labor market. It is argued that if Australia's labor force participation rate were not adjusted, inflation would expand, while recessions would be more deflationary and lead to a larger increase in involuntary unemployment. Elshamy (52) used annual data from International Financial Statistics (IFS) and cointegration analysis for the period 1970-2010 to estimate the long-term Okun coefficient for Egypt. The results showed that the coefficients are statically significant under the expected sign when Okun's Law is estimated in Egypt in the long and short term. Khrais and Al-Wadi (53) used the period 1990-2016 and simple linear regression to analyze the relationship between GDP growth and unemployment in MENA countries. The results showed that no significant effect is observed on the total GDP, which is representative of all countries participating in the unemployment study, calculated based on the size of the labor force in these countries. Hjazeen et al. (54) used an autoregressive distributed lag (ARDL) model to investigate the impact of unemployment on the Jordanian economy during the period 1991-2019. Their findings show a negative association between economic growth and unemployment, and a positive relationship between educations, female and urban populations, and unemployment in Jordan.

## Data and methods

### Data

**Figure 3:**
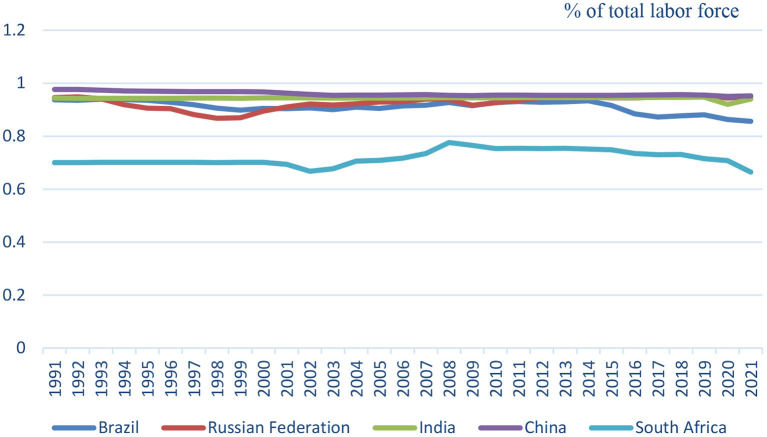
1991-2021 BRICS employment rate.

We use per capita of GDP in this paper, which is GDP per capita (constant 2010 US$). Health expenditure per capita (Current of US$) is used for health expenditure. The employment rate is calculated using unemployment, total (percentage of the total labor force), and then use one minus unemployment rate, then we can get employment rate (see Figure 3). The data comes from public information of the World Bank Data. Because the data are limited to health expenditure which is the year from 2000 to 2019, the data period of GDP and employment rate, we use also from 2000 to 2019, totaling 20 years of annual data.

### Methods

**Figure 4:**
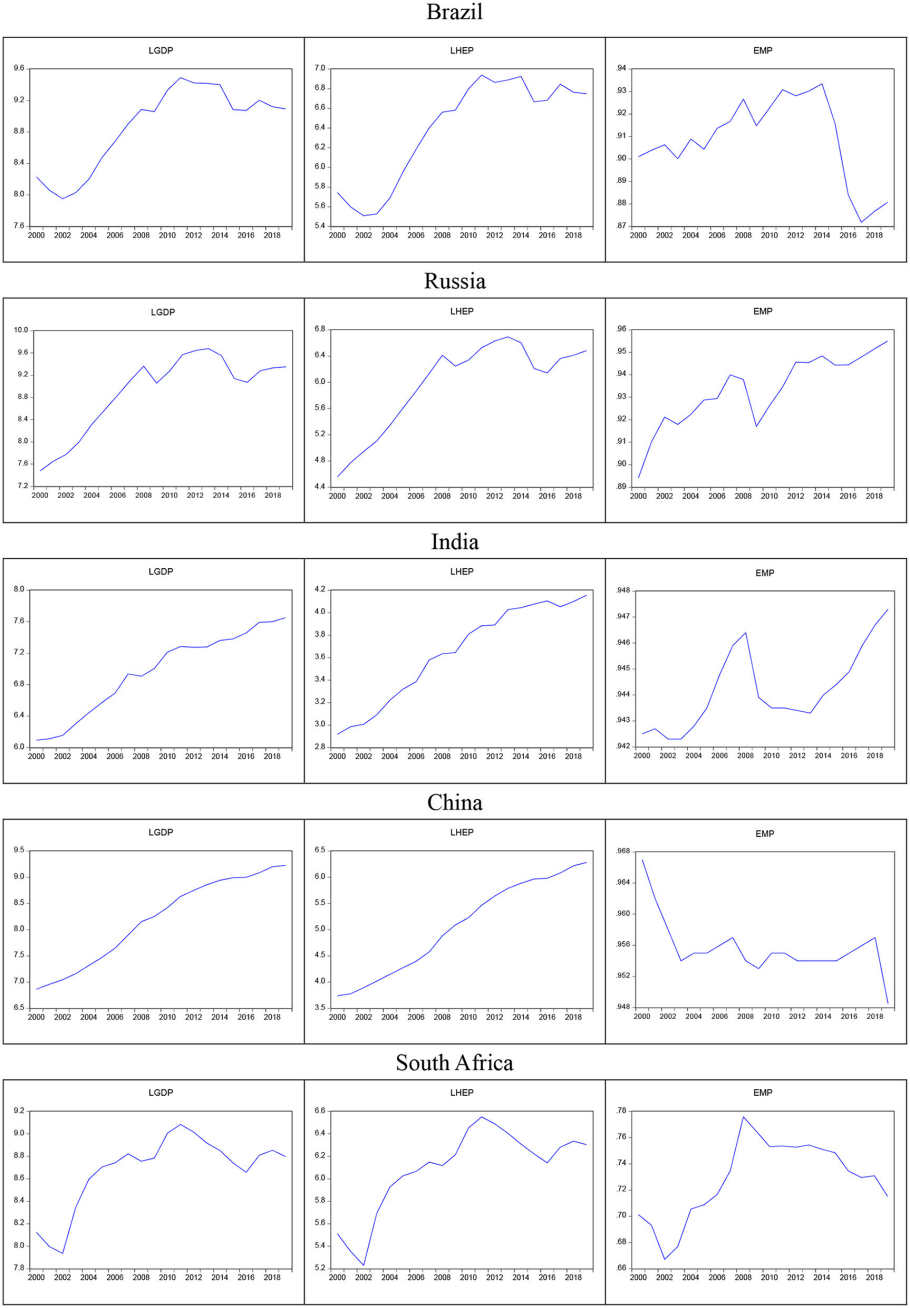
BRICS 2000-2019 GDP, health expenditure and employment rate.

We use the Fourier Bootstrap ARDL Model to examine the nexus test the health expenditure, economic growth, and employment rate relationship of BRICS countries; the Fourier Bootstrap ARDL Model uses the principle of self-regression and multiple loop calibrations to make the time-series related data close to the expected result that needs to be verified. Before doing the Bootstrap autoregressive distribution lag model, it is necessary to know whether the collected data is for the fixed state, the general treatment method is the unit root test first. The purpose of a unit root test is to determine the integration level of time-series variables to determine the nature of the time-series. The Fourier Bootstrap ARDL model uses the fundamental of autoregression and multi-circulation calibration to bring the data related to the time-series close to the expected results that need to be verified. It is also a kind of method of Monte Carlo simulation. The sample period of the time-series data is short (20 years), the results of the verification through the interaction of the lag period, and 5,000 repeated circulations. However, the results of this kind of Bootstrap loop calculation, although the period of the time-series is short, will not affect its validity. Using the Fourier Bootstrap ARDL test model, we can better understand the cointegration state of the time-series in the model and use Monte Carlo simulations to determine the size and power of the endogenous problem framework. The simulated asymptotic threshold has little effect endogenous problem framework. The simulated asymptotic threshold has little effect. If the re-sampling process is appropriately applied, the pilot test ratio can be determined, and progressive testing in ARDL testing based on size and power characteristics can be performed and eliminated more appropriately (see Figure 4).

According to the papers of Gallant (55) and Gallant and Souza (56), they pointed out that a small number of low frequency components of Fourier approximation can capture an unknown number of progressive and sharp structural breaks. Yilanci et al. (57) used Fourier Bootstrap with sharp breaks (using the Dummy variable). Fourier Bootstrap ARDL is modified to Bootstrap ARDL with sharp breaks to smooth break (using Fourier function). Pesaran et al. (58) and the subsequent Fourier Bootstrap ARDL model, we can write the following ARDL Bounds test model:


(1) $$\begin{array}{l} \Delta Y_{t}=c+\alpha Y_{t-1}+\beta X_{t-1}+\Sigma_{i=1}^{p-1}{\theta \Delta Y}_{t-i}+\Sigma_{i=1}^{p-1}\delta X_{t-i}\\ \;+\Sigma_{j=1}^{q}\eta D_{t,j}+\varepsilon_{t} \end{array}$$

The above equation does not require feedback from Y to X. This means that we cannot allow two or more variables (weak) to be endogenous, and the researchers ignored this assumption in the empirical significance of Pesaran et al. (58) ARDL Bounds test. This does not exclude the cointegration relationship between regressions, nor does it assume that there is no (short-term) Granger (59) causality between the dependent variable and the regression. It assumes that the regression variable is weakly exogenous. In the long-term, these regression variables are not affected by the dependent variable, but this does not exclude the cointegration relationship between the regressions, nor does it assume that there is no (short-term) Granger (59) causality between the regression and the dependent variable. The researchers ignored this assumption in the empirical implications of the ARDL Bounds test.

According to the research of Pesaran et al. (58), for the following hypotheses, the cointegration test requires *F*-test or *t*-test:


$$\begin{array}{l} H_{0}:\;\alpha =\beta =0\;or\;H_{0}:\alpha =0 \end{array}$$

McNown et al. (45) suggested supplementing the existing *F*-test and *t*-test for cointegration proposed by Pesaran et al. (58) by adding an additional *F*_2_ test. McNown et al. (45) improved the ARDL Bounds test of Pesaran et al. (58) and used the Bootstrap ARDL method test to distinguish all three defined cointegration, non-cointegration, and degeneration cases. They set the degeneration cases as follows:

1. When the test result is significant in the *F*-test and *t*-test of the lagged independent variable, and the *F*_2_ test of the lagged dependent variable is not significant, it is degeneration situation #1.
2. When the test result is significant in the *F* test and *t* test of the lagged dependent variable, but the lagged independent variable is not significant, it is degeneration case #2.

Pesaran et al. (58) proposed a critical value for degeneration case #2, but there is no critical value for degeneration case #1. To exclude degeneration case #1, the order of integration of the dependent variable must be *I*(1) but Perron (60) thought the unit root test is un-reliable for its low degree of inspection.

The advantage of the Bootstrap ARDL test is that the endogenous problem has no effect on the size and power characteristics of the ARDL Bounds test frame. Using the asymptotic threshold of Monte Carlo simulation can help solve this problem. Another feature is that it can solve the sample on the time-series. The problem of insufficient in the World Bank database, only 20-year samples of health expenditure (2000-2019) can be obtained, and the Bootstrap ARDL test can solve this kind of problem efficiently. McNown et al. (45) also proposed an extension of the Bootstrap ARDL test framework for alternative degeneration scenarios, with a threshold generated by the Bootstrap ARDL test.

Therefore, the recommended the Bootstrap ARDL test can better understand the cointegration status of the time-series in the model. Yilanci et al. (57) followed Becker et al. (61) and Ludlow and Enders (62) and allowed the use of single frequency. For example, we want to use GDP as a control variable to test whether workforce employment affects health expenditures, and then the model can be expressed as:


(2) $$\begin{array}{l} HEP_{t}=\alpha_{0}+\alpha_{1}GDP_{t}+\alpha_{2}EMP_{t}+\varepsilon_{t} \end{array}$$

*HEP* is health expenditure, *GDP* is gross domestic production, *EMP* is workforce employment, and Equation (2) is extended to the Bootstrap ARDL situation. The transform model is following as:


(3) $$\begin{array}{l} \Delta HEP_{t}=\beta_{0}+\beta_{1}HEP_{t-1}+\beta_{2}GDP_{t-1}+\beta_{3}EMP_{t-1}\\ \;+\Sigma_{i=1}^{p-1}\varphi_{i}^{\prime }\;\Delta HEP_{t-i}+\Sigma_{i=1}^{p-1}\delta_{i}^{\prime }\;\Delta GDP_{t-i}\\ \;+\Sigma_{i=1}^{p-1}\emptyset_{i}^{\prime }\;\Delta EMP_{t-i}+e_{t} \end{array}$$

(4) $$\begin{array}{l} H_{0A}:\beta_{1}=\beta_{2}=\beta_{3}=0 \end{array}$$

(5) $$\begin{array}{l} H_{0B}:\beta_{1}=0 \end{array}$$

(6) $$\begin{array}{l} H_{0C}:\beta_{2}=\beta_{3}=0 \end{array}$$

The above model, Equation (3), can also incorporate dummy variables to capture structural breakpoints *D*_*t*_. We can think of this model as a Bootstrap ARDL with obvious structural sharp breakpoints. Since Gallant (55) and Gallant and Souza (56) showed that a small amount of low-frequency components of the Fourier approximation can capture an unknown number of progressive and sharp breakpoints, we use the following Fourier function to replace the dummy variables method:


(7) $$\begin{array}{l} d\left(t\right)=\Sigma_{k=1}^{n}\alpha_{k}(sin\frac{2\pi kt}{T})+\Sigma_{k=1}^{n}b_{k}\cos\left(\frac{2\pi kt}{T}\right)\; \end{array}$$

(8) $$\begin{array}{l} d\left(t\right)=\gamma_{1}\left(\sin\frac{2\pi kt}{T}\right)+\gamma_{2}\cos\left(\frac{2\pi kt}{T}\right) \end{array}$$

The Fourier Bootstrap ARDL model is estimated as follows:


(9) $$\begin{array}{l} \Delta HEP_{t}=\beta_{0}+\gamma_{1}(sin\frac{2\pi kt}{T})+\gamma_{2}\cos\left(\frac{2\pi kt}{T}\right)+\beta_{2}HE_{t-1}\\ \;+\beta_{3}GDP_{t-1}+\beta_{4}EMP_{t-1}+\Sigma_{i=1}^{p-1}\alpha_{i}^{\prime }\;\Delta HEP_{t-i}\\ \;+\Sigma_{i=1}^{p-1}\delta_{i}^{\prime }\;\Delta GDP_{t-i}+\Sigma_{i=1}^{p-1}\emptyset_{i}^{\prime }\Delta EMP_{t-i}+e_{t}\; \end{array}$$

Following the research of Christopoulos and Leon-Ledesma (63) and Omay (64), we use all values of *k* in the interval of *k* = [0.1, ..., ..., 5] to estimate Equation (9), which The increment is 0.1 and the *k* that produces the smallest residual sum of squares is selected. Christopoulos and Leon-Ledesma (63) proposed that structural frequencies represent permanent breakpoints, while integer frequencies represent temporary breakpoints. Fourier ARDL's model, the same as Bootstrap ARDL model, is to test the long-term cointegration relationship of variables; when there is cointegration, it will test whether there is a causal relationship. When there is no cointegration, short-term Granger causality between variables is tested. Therefore, we use Bootstrap ARDL to estimate the critical values of $F_{1}^{*}$, $F_{2}^{*}$ and t^*^ and Fourier frequency (smooth) to transfer the breakpoints. A detailed description of the Bootstrap ARDL can be found in the paper by McNown et al. (45).

## Empirical results and discussion

### Empirical results

Table 2 shows the statistical description data of the gross national production, health expenditure, and employment rate of the BRIC countries. From the skewness point of view, only India's and China's employment rate show a long-tailed to the left (positive value). For the rest of skewness of the BRICS's countries, it shows a long-tailed state (negative value) to the right. From the perspective of kurtosis, the BRICS countries both show low kurtosis in terms of gross national product, health expenditure, and employment rate. China's employment rate (6.401427) is high and narrow of bell- shaped distribution. South Africa's GDP (3.329396) and health expenditure (3.109702) approaches the standard kurtosis (=3). The results of the entire Jarque-Bera test, China's employment rate (*P*-value = 0.000265) and South Africa's GDP (*P*-value = 0.096888) approach to the standard normal distribution (*P* < 10%).

**Table 2:** Description of statistics.

**Economies**	**Brazil**			**Russia**		
**variables**	**LGDP**	**LHEP**	**EMP**	**LGDP**	**LHEP**	**EMP**
Mean	8.865502	6.393775	0.908515	8.903412	5.969350	0.933120
Median	9.078165	6.623862	0.911250	9.127668	6.228269	0.936250
Max	9.491405	6.938843	0.933400	9.678758	6.693419	0.955000
Min	7.951380	5.509174	0.871800	7.479635	4.558205	0.894200
Std. Dev	0.520298	0.524114	0.018621	0.695221	0.671408	0.015840
Skewness	−0.592940	−0.643664	−0.542732	−0.872967	−0.888673	−0.700231
Kurtosis	1.887786	1.786890	2.288053	2.420230	2.390982	2.864377
Jarque-Bera	2.202777	2.607374	1.404249	2.820348	2.941550	1.649742
Probability	0.332409	0.271529	0.495531	0.244101	0.229747	0.438292
Observations	20	20	20	20	20	20
**Economies**	**India**			**China**		
**variables**	**LGDP**	**LHEP**	**EMP**	**LGDP**	**LHEP**	**EMP**
Mean	6.965644	3.646615	0.944200	8.193605	5.066531	0.955675
Median	7.109147	3.727540	0.943700	8.337093	5.159893	0.955000
Max	7.650050	4.154943	0.947300	9.224622	6.282516	0.967000
Min	6.094279	2.917900	0.942300	6.866279	3.740342	0.948500
Std. Dev	0.527199	0.429951	0.001535	0.828711	0.896340	0.003664
Skewness	−0.423433	−0.422447	0.588830	−0.307180	−0.142820	1.431598
Kurtosis	1.802818	1.703925	2.168510	1.585487	1.503293	6.401427
Jarque-Bera	1.792023	1.994714	1.731880	1.981903	1.934768	16.4730***
Probability	0.408195	0.368853	0.420656	0.371223	0.380076	0.000265
Observations	20	20	20	20	20	20
**Economies**	**South Africa**					
**variables**				**LGDP**	**LHEP**	**EMP**
Mean				8.677335	6.089227	0.728420
Median				8.770447	6.181502	0.732750
Max				9.083748	6.551034	0.775900
Min				7.936334	5.229939	0.667100
Std. Dev				0.326120	0.372513	0.029662
Skewness				−1.171919	−1.043917	−0.448140
Kurtosis				3.329396	3.109702	2.333722
Jarque-Bera				4.668401*	3.642569	1.039370
Probability				0.096888	0.161818	0.594708
Observations				20	20	20

The descriptive statistics are based on the differences in the logarithms of each variable except EMP. The asterisks ***, ** and * indicate the 1, 5, and 10% significance levels.

Tables 3, 4 show the results of the unit root test for the stationary of the three variable time-series data. The unit root test is divided into the level item *I*(0) test (Table 3) and the first-order difference item *I*(1) test (Table 4). The three variables only satisfy *I*(0) or *I*(1) test can use Fourier ARDL for cointegration test. The test results show that the three variables of the five countries meet the standard, so the time-series data is stationary. In addition, after the unit root test is completed, the Akaiki Information Criteria (AIC) needs to be determined. In this paper, we use the AIC criteria to select and judge the smallest AIC as the best lag period. We used three DF, ADF, and PP unit root tests. Among them, DF is used for Intercept and Trend and Intercept, while ADF and PP are used for Intercept, Trend Intercept, and no intercept and trend verification. Table 3 is for the level item *I*(0) test; Table 4 is the level item *I*(1) test. According to McNown et al. (45), it is pointed out that Bootstrap ARDL has no unit as long as it satisfies *I*(0) or *I*(1). Root, then the cointegration test can be performed. In Table 3, the three variables *I*(0) unit root tests of the BRICS countries, most of the test results are unstable series. Remember that we did the *I*(1) unit root test in Table 4, and most of them showed a stationary series.

**Table 3:** Unit root test (Level term).

**Countries**	**Test**	**DF**		**ADF**			**PP**		
	**variable**	**Intercept**	**Trend and intercept**	**Intercept**	**Trend and intercept**	**None**	**intercept**	**Trend and intercept**	**None**
Brazil	LGDP	−1.6261* (1)	−1.5856 (1)	−2.5621 (2)	−0.4815 (0)	1.1538 (0)	−1.1956 (1)	−0.7570 (1)	0.8965 (2)
	LHEP	−1.4804 (1)	−1.6437 (1)	−2.3355 (2)	−0.6210 (1)	1.4960 (0)	−1.0940 (2)	−0.9137 (1)	1.1760 (2)
	EMP	−1.5872 (1)	−1.7831 (1)	−1.5114 (1)	−1.0504 (0)	−0.4451 (0)	−1.0802 (1)	−1.0504 (0)	−0.4013 (1)
Russia	LGDP	−1.2273 (1)	−1.1521 (1)	−2.4703 (0)	−1.0406 (0)	1.8782 (0)	−2.3939 (2)	−1.0653 (2)	1.5992 (1)
	LHEP	−1.0409 (1)	−1.6028 (1)	−2.7337* (0)	−1.1452 (0)	0.8982 (1)	−2.5292 (1)	−1.2241 (1)	1.6400 (2)
	EMP	−1.4561 (0)	−3.2913* (1)	−2.4184 (0)	−3.6616* (0)	1.7061 (0)	−2.4363 (2)	−3.6745** (2)	1.8711 (2)
India	LGDP	−0.6147 (1)	−1.5069 (1)	−2.6558 (2)	−1.0581 (0)	4.5807 (0)	−1.2685 (0)	−1.0581 (1)	4.4358 (1)
	LHEP	−0.3840 (1)	−0.9918 (1)	−1.7195 (0)	0.7818 (2)	4.3019 (0)	−2.0091 (2)	−0.2679 (2)	4.1651 (2)
	EMP	−1.4405 (1)	−2.4725 (1)	−1.3404 (1)	−2.2123 (1)	1.3363 (0)	−0.8394 (1)	−1.5639 (1)	1.1580 (1)
China	LGDP	−1.0826 (1)	−1.2442 (1)	−1.8694 (0)	0.3825 (0)	0.0463 (1)	−1.5205 (2)	−0.1652 (2)	5.0038 (2)
	LHEP	−1.1118 (1)	−2.8859 (1)	−1.5774 (0)	−0.9850 (0)	1.496677 (1)	−0.6567 (2)	−1.1460 (2)	5.2849 (2)
	EMP	−2.0458** (0)	−3.0512* (0)	−3.1755** (0)	−3.5159* (1)	−1.5766 (0)	−3.8223** (2)	−3.9339** (2)	−1.5766 (0)
South Africa	LGDP	−2.2495** (1)	−2.3365 (1)	−3.0893** (1)	−2.9968 (2)	0.8268 (1)	−1.7617 (0)	−1.1239 (0)	1.0103 (0)
	LHEP	−2.0022** (1)	−2.2527 (1)	−2.6609* (1)	−2.7118 (2)	0.9497 (1)	−1.5263 (0)	−1.2362 (2)	0.9413 (1)
	EMP	−1.2191 (0)	−1.0359 (0)	−1.2742 (0)	−0.7261 (0)	0.1539 (0)	−1.3773 (1)	−0.9347 (1)	0.1307 (1)

GDP represents logarithms of Gross Domestic Product per capita; HEP represents logarithms of health expenditure per capita; EMP represents employment rate; the parentheses are optimal lag order based on Akaike Information Criterion (AIC). The asterisks ^***^, ^**^ and ^*^ indicate the 1, 5, and 10% significance levels. The numbers in parentheses represent the lag period. As an empirical test, we first performed Dickey-Fuller (DF) (32), Augmented Dickey-Fuller (ADF) (32), and Phillips and Perron (PP) (65) tests on the unit root hypothesis among variables.

**Table 4:** Unit root test (1^st^ difference term).

**Countries**	**Test**	**DF**		**ADF**			**PP**		
	**variable**	**Intercept**	**Trend and intercept**	**Intercept**	**Trend and intercept**	**None**	**Intercept**	**Trend and intercept**	**None**
Brazil	LGDP	−2.6123** (0)	−3.157064* (0)	−2.8058* (0)	−3.2214 (0)	−2.6059** (0)	−2.7554* (2)	−3.2214 (2)	−2.6059** (0)
	LHEP	−2.6322** (0)	−3.0949* (0)	−2.8423* (0)	−3.2184 (2)	−1.8437* (2)	−2.8749* (2)	−3.1498* (2)	−2.4520** (2)
	EMP	−3.0819*** (0)	−3.3547** (0)	−3.0220* (0)	−2.7216 (0)	−2.5156** (0)	−3.0220** (0)	−3.0890 (2)	−3.0820*** (0)
Russia	LGDP	−2.8805*** (0)	−3.5321** (0)	−2.8019* (0)	−3.4963* (1)	−2.5656** (0)	−2.7624* (2)	−3.2692* (2)	−2.5116** (1)
	LHEP	−2.5926** (0)	−3.1703* (0)	−2.5614 (0)	−3.4964* (2)	−2.2962** (0)	−2.6005 (1)	−2.9681 (2)	−2.2316** (2)
	EMP	−3.7924*** (0)	−4.1937*** (0)	−4.2392*** (0)	−4.0616** (0)	−4.0207*** (0)	−4.3846*** (2)	−4.1443** (2)	−4.0967*** (2)
India	LGDP	−3.8994*** (0)	−4.5023*** (0)	−3.9852*** (0)	−4.4598** (1)	−1.3860 (1)	−3.9831*** (1)	−4.4431** (1)	−2.0032** (1)
	LHEP	−4.1928*** (0)	−4.9790*** (1)	−4.7549*** (1)	−4.7341*** (0)	−2.2891** (0)	−4.0674*** (1)	−4.9901*** (2)	−2.1002** (2)
	EMP	−2.8984*** (0)	−2.9751* (0)	−2.8096* (0)	−2.7953 (0)	−2.6887* (0)	−2.8394* (1)	−2.8309 (1)	−2.7060*** (2)
China	LGDP	−1.8558* (0)	−2.5355 (0)	−1.7955 (0)	−2.6043* (1)	−1.0011* (0)	−1.7955 (0)	−2.4134* (0)	−1.9640* (1)
	LHEP	−2.2473** (0)	−2.6642 (0)	−0.7876 (1)	−2.6474* (0)	−0.8657 (0)	−2.6606* (2)	−0.8180 (2)	−2.6474 (1)
	EMP	−2.0546** (0)	−2.4154 (0)	−2.4717 (0)	−1.6281 (1)	−2.4180** (1)	−2.3997 (1)	−1.4580 (0)	−2.2013** (1)
South Africa	LGDP	−2.7926*** (0)	−4.3464*** (1)	−2.9311* (0)	−5.1593*** (1)	−2.7970*** (0)	−2.9817* (1)	−3.4395* (0)	−2.8631*** (1)
	LHEP	−2.9723*** (0)	−4.2645*** (1)	−3.1755** (0)	−5.4195*** (1)	−2.9691*** (0)	−3.1862** (1)	−3.5466* (0)	−2.9690*** (0)
	EMP	−3.1020*** (0)	−3.5077** (0)	−3.1032** (0)	−4.9963*** (1)	−3.1873*** (0)	−3.1031** (1)	−3.2898* (1)	−2.9690*** (0)

GDP represents logarithms of Gross Domestic Product per capita; HEP represents logarithms of health expenditure per capita; EMP represents employment rate; the parentheses are optimal lag order based on Akaike Information Criterion (AIC). The asterisks ^***^, ^**^ and ^*^ indicate the 1, 5, and 10% significance levels. The numbers in parentheses represent the lag period. As an empirical test, we first performed Dickey-Fuller (DF) (32), Augmented Dickey-Fuller (ADF) (32), and Phillips and Perron (PP) (65) tests on the unit root hypothesis among variables.

Table 5 shows three variables of economic fluctuation, workforce employment, and health expenditure of the BRICS countries. The data period is 2000-2018, Fourier frequency, lag term, and dummy variables. The late-term is selected by the VAR method, and the dummy variable or breakpoint is selected by Bai and Perron (66). From Table 5, we can see that Russia uses Economic Fluctuation as the dependent variable and workforce employment and health expenditure as the independent variable. When the Fourier frequency is 5, it means that the relative changes of these three variables are relatively smooth and gentle (the larger the frequency number, the frequency span the longer).

**Table 5:** Fourier frequency and sharp breakpoints.

**Country**	**Variables**	**Period**	**Fourier frequency**	**Lag specification**	**Sharp breakpoints**
Brazil	LGDP | LHEP | EMP	2000–2019	3.1	(0, 0, 0) (0, 0, 0) (0, 1, 1)	D07 | D07 | D07, D16
	LHEP | EMP | LGDP	2000–2019	3.0	(0, 0, 0) (0, 1, 1) (0, 0, 0)	D07 | D07, D16 | D07
	EMP | LGDP | LHEP	2000–2019	3.2	(0, 1, 1) (0, 0, 0) (1, 0, 0)	D07, D16 | D07 | D07
Russia	LGDP | LHEP | EMP	2000–2019	2.1	(0, 0, 0) (0, 0, 0) (1, 0, 0)	D03, D06 | D03, D06 | D04, D12
	LHEP | EMP | LGDP	2000–2019	2.0	(0, 0, 0) (1, 0, 0) (0, 0, 0)	D03, D06 | D04, D12 | D03, D06
	EMP | LGDP | LHEP	2000–2019	1.1	(1, 0, 0) (0, 0, 0) (0, 0, 0)	D04, D12 | D03, D06 | D03, D06
India	LGDP | LHEP | EMP	2000–2019	1.9	(1, 0, 0) (0, 0, 0) (1, 0, 0)	D04, D07, D10, D16 | D04, D07, D11 | D05, D17
	LHEP | EMP | LGDP	2000–2019	2.7	(0, 0, 0) (1, 0, 0) (1, 0, 0)	D04, D07, D11 | D05, D17 | D04, D07, D10, D16
	EMP | LGDP | LHEP	2000–2019	1.8	(1, 0, 0) (1, 0, 0) (0, 0, 0)	D05, D17 | D04, D07, D10, D16 | D04, D07, D11
China	LGDP | LHEP | EMP	2000–2019	0.9	(1, 0, 2) (0, 0, 1) (0, 0, 1)	D05, D08, D12 | D05, D09, D13 | D03
	LHEP | EMP | LGDP	2000–2019	0.1	(0, 0, 1) (0, 0, 1) (1, 0, 2)	D05, D09, D13 | D03 | D05, D08, D12
	EMP | LGDP | LHEP	2000–2019	0.6	(0, 0, 1) (1, 0, 2) (0, 0, 1)	D03 | D05, D08, D12 | D05, D09, D13
South Africa	LGDP | LHEP | EMP	2000–2019	1.0	(0, 0, 2) (0, 2, 0) (0, 0, 0)	D04 | D04, D9 | D07, D16
	LHEP | EMP | LGDP	2000-2019	1.0	(0, 2, 0) (0, 0, 0) (0, 0, 2)	D04, D09 | D07, D16 | D04
	EMP | LGDP | LHEP	2000–2019	2.9	(0, 0, 0) (0, 0, 2) (0, 2, 0)	D07, D16 | D04 | D04, D09

Table 6 shows the cointegration test results of the three variables of economic fluctuation, workforce employment and health expenditure in the BRICS countries. We find that China among the five BRICS countries has a cointegration relationship whether it is based on health expenditure as dependent variable, and economic fluctuation, workforce employment rate as the independent variable. economic fluctuation, workforce employment and health expenditure of Brazil, Russia, India and South Africa have no cointegration relationship; but India's economic fluctuation and workforce employment as dependent variable and the rest of variable as an independent variable, which shows degeneration case #1 described by McNown et al. (45), the case also shows on Economic Fluctuation as dependent variable of China.

**Table 6:** Cointegration analysis.

**Countries**	**Period**	**Dependent variable | independent variable**	**Fourier frequency**	**AIC**	***F***	F*	**t_1_**	t${\;}_{1}^{*}$	**t_2_**	t${\;}_{2}^{*}$	**Cointegration status**
Brazil	2000–2019	LGDP | LHEP | EMP	3.1	−4.610496	1.475	4.539	0.397	−1.006	0.545	5.262	No-cointegration
	2000–2019	LHEP | EMP | LGDP	3.0	−1.264442	0.826	11.628	−0.765	0.729	0.291	16.745	No-cointegration
	2000–2019	EMP | LGDP | LHEP	3.2	−7.338889	1.361	7.860	−1.988	0.533	1.709	4.220	No-cointegration
Russia	2000–2019	LGDP | LHEP | EMP	2.1	−3.760923	2.107	4.973	−0.073	−1.984	0.393	5.206	No-cointegration
	2000–2019	LHEP | EMP | LGDP	2.0	−4.231599	2.185	7.031	−0.888	−1.553	0.317	4.093	No-cointegration
	2000–2019	EMP | LGDP | LHEP	1.1	−7.933311	4.442	9.638	−3.313	−4.579	3.676	8.509	No-cointegration
India	2000–2019	LGDP | LHEP | EMP	1.9	−3.181801	10.797	7.505	−3.791	−2.955	6.377	8.135	Degeneration Case #1
	2000–2019	LHEP | EMP | LGDP	2.7	−3.611721	8.056	8.383	−4.897	−3.574	6.199	9.866	No-cointegration
	2000–2019	EMP | LGDP | LHEP	1.8	−11.76839	8.128	8.056	−4.897	−3.580	6.199	9.867	Degeneration Case #1
China	2000–2019	LGDP | LHEP | EMP	0.9	−4.841960	8.412	8.043	−1.662	0.733	0.791	3.754	Degeneration Case #1
	2000–2019	LHEP | EMP | LGDP	0.1	−4.500331	13.347	3.390	−5.810	−2.068	19.038	3.236	Cointegration
	2000–2019	EMP | LGDP | LHEP	0.6	−10.18717	4.091	7.953	−2.600	−4.082	2.721	10.143	No-cointegration
South Africa	2000–2019	LGDP | LHEP | EMP	1.0	−4.764121	3.300	41.684	0.679	−0.864	0.754	20.123	No-cointegration
	2000–2019	LHEP | EMP | LGDP	1.0	−4.468249	6.098	4.311	1.652	−1.350	3.391	4.360	Degeneration Case #1
	2000–2019	EMP | LGDP | LHEP	2.9	−6.199231	3.609	5.778	−2.685	−3.357	5.337	6.873	No-cointegration

GDP, logarithms of Gross Domestic Product per capita; HEP, logarithms of health expenditure per capita; EMP, employment rate; F_1_ is the F statistic for the coefficients of y(-1), x_1_(-1) and x_2_(-1); F_2_ is the F statistic for the coefficients of x_1_(-1) and x_2_(-1); t denotes the t statistic for the coefficient of y(-1). D04 means a dummy variable for the year 2004; other years are 0. t is the t-statistics for the dependent variable, and F is the F-statistics for the independent variable. F , t_1_, t_2_ and F^*^ t${\;}_{1}^{*}$, t${\;}_{2}^{*}$ are the critical values at the 10% significance level, generated from the bootstrap method suggested by McNown et al. (45).

In addition, according to McNown et al. (45), we test all the cointegration variables in China to see whether there is a causality relationship in the long-term. We find that there is no any significant causality relationship (see Table 7).

**Table 7:** Causality test (Long-term).

**Country**		**LGDP**	**LHEP**	**EMP**
		**F-statistics (*P*-value)**	**F-statistics (*P*-value)**	**F-statistics (*P*-value)**
China	LHEP	0.767126/[0.3983]	/	0.875857/[0.4553]

GDP, logarithms of Gross Domestic Product per capita; HEP, logarithms of health expenditure per capita; EMP, employment rate; The asterisks ^***^, ^**^ and ^*^ indicate the 1%, 5% and 10% significance levels. Additionally, the parentheses [.] are p-value and sign for the coefficients. The case of non-cointegration and its causality test involved only lagged differenced variables. Bold value represents Granger causality results.

In the short-term Granger causality test (see Table 8), our research find that economic fluctuations in Brazil (29.21084), China (45.42108), and South Africa (5.287764) have a positive effect on the one-period lag of health expenditure. The employment rate has a negative effect on health expenditures in Brazil (39.00020). In the mid-2010s, especially at the end, the unemployment rate basically coincided with the economic cycle stage. Especially since 1995, the employment situation has become increasingly severe, which is consistent with the continued decline in economic growth. The level of unemployment fluctuates with changes in the economic cycle stage, indicating that China has experienced obvious cyclical unemployment. The employment rate has a positive effect on China's (8.087294) health expenditure.

**Table 8:** Causality test.

**Countries**		**LGDP**	**LHEP**	**EMP**
		**F- statistics (P-value)**	**F- statistics (*P*-value)**	**F- statistics (*P*-value)**
Brazil	LGDP	/	3.995751*/[0.0767]	9.627105**/[0.0127]
	LHEP	29.21084***/[0.0000]	/	39.00020***/[0.0000]
	EMP	5.906825**/[0.0380]	5.323693**/[0.0464]	/
Russia	LGDP	/	29.46711***/[0.0006]	1.349793/[0.1656]
	LHEP	1.606092/[1.3295]	/	2.490945/[0.1490]
	EMP	0.004573/[0.4976]	0.452017/[0.5183]	/
India	LGDP	/	2.729393*/[0.0985]	2.303124*/[0.0912]
	LHEP	1.434677/[0.2653]	/	2.818042/[0.1515]
	EMP	15.08583***/[0.0005]	5.415685*/[0.0667]	/
China	LGDP	/	2.010121/[0.1940]	0.581622/[0.4676]
	LHEP	45.42108***/[0.0000]	**/**	8.087294**/[0.00373]
	EMP	0.925015**/[**0.3613**]**	0.593607**/[**0.4999**]**	/
South Africa	LGDP	/	7.905611**/[0.0184]	12.64342***/[0.0052]
	LHEP	5.287764*/[0.0505]	/	0.086489/[0.7762]
	EMP	0.685179/[0.2083]	0.949613/[0.2053]	/

GDP, logarithms of Gross Domestic Product per capita; HEP, logarithms of health expenditure per capita; EMP, employment rate; The asterisks ^***^, ^**^ and ^*^ indicate the 1, 5, and 10% significance levels. Additionally, the parentheses [.] are p-value and sign for the coefficients. The case of non-cointegration and its causality test involved only lagged differenced variables. Bold value represents Granger causality results.

Economic fluctuations in Brazil (5.906825) have a positive effect on the employment rate. Although the Brazilian economy achieved positive growth in 2018 compared with the previous year, factors such as high unemployment, weak economic growth, and uncertain prospects for economic reform have brought uncertainty to the future development of the Brazilian economy. Health expenditures in Brazil (3.995751), Russia (29.46711) and India (2.729393) all have a negative effect on economic fluctuations, while South Africa (7.905611) has a positive effect. Health expenditures in Brazil (5.323693) and India (5.415685) have a lagging negative effect on the employment rate. The employment rates of Brazil (9.627105) and South Africa (46.23093) have a significant negative effect on economic fluctuations, while India (12.64342) has a positive impact. Brazil's workforce employment rate and health expenditure are negatively causally related to each other. Brazil's unemployment rate began to increase in 2015, and the economic fluctuations were fluctuating; Russia's short-term health expenditure has a significant negative effect on GDP. India's short-term workforce employment rate and GDP have a positive causal relationship with each other.

### Discussion

Implementing universal health coverage will help eliminate this cause of poverty (67). In the new decree issued in 1988, Brazil clarified the reform concept of “universal coverage, fairness, continuity, and integration” of the health care system, and established “decentralization” and “state and city government as the main body” reforms in principle, the United Health System (SUS) (68) was created. The new decree holds that the state and the government have a responsibility to provide free medical services to every citizen, and protecting the health of citizens is an important responsibility of the state and the government. Within the unified health system, everyone is equal, and system services from preventive health care, disease diagnosis, and treatment to rehabilitation care can be provided to meet the different medical needs of different populations. Faced with a serious national crisis in 2000, Putin, Russian President, put forward the concept of reform of the new government, claiming to strengthen the ability of the country to govern, focusing on economic development, alleviating social contradictions, and achieving the goal of building a strong state. The Russian government has initiated comprehensive reforms to promote the ministry, seeking to reduce the number of government functions (69). The Ministry of Social Development and the Ministry of Health was abolished and merged into the Ministry of Health Care and Social Development to assume social development and health care functions so that a wider range of departments were involved in solving health problems and greatly increased coordination.

During this period, China adopted a conservative international financial policy and strict foreign exchange controls. Therefore, it should not have been affected by the 1997, Asian financial crisis and the 2008 subprime mortgage crisis. Its economic changes still maintain a high economic growth rate. From the experience of developed countries, full employment represents the equilibrium state of the economy. China has made full use of the government's abundant financial opportunities in recent years to seek effective social security measures to eliminate the negative impact of expected changes in system reforms on consumption, so that urban and rural residents have the courage to consume without worries. If you have money, dare to consume, and continue to enhance consumer confidence.

When India first established the country, it imitated the socialist value system of the former Soviet Union and established a medical insurance system with a small coverage. It was the first medical insurance system in Asia. Due to the serious shortage of government financial investment in public free medical services, the level and quality of medical services provided by public medical and health institutions are very poor. Many patients have to choose private hospitals for treatment, which has promoted the rapid development of private hospitals. After the implementation of the new industrial policy in India, privatization reforms in the economic system have also promoted privatization reforms in the medical and health field, spurring the rapid expansion of private medical institutions. As the number of private medical institutions continues to rise, they have basically monopolized cities and cities. The vast majority of outpatient services in rural areas (70). In 2003, the transformation of China's medical and health system was closely linked to the reform of the national economic system, and it experienced two medical and health system reforms. In the process of the government's continuous promotion of economic system reform, in order to adapt to the development of the socialist market economic system, the reform of the medical system has also undergone a process of continuous development and deepening.

Start by adjusting the prices of medical services and medicines, and gradually develop to comprehensive reform. Since 2007, the Chinese government has gradually pushed forward the reform of the new medical and health system. The new medical reform has furthered the reform of the medical system with the orientation of advocating government leadership and public welfare. According to WHO data, private voluntary health insurance expenditure in South Africa accounts for 42% of total health expenditure, ranking first in the world. However, private health insurance only covers 16% of the South African population. In South Africa, different socioeconomic groups have unequal access to health care (71), and South Africa has now introduced a national health insurance system that provides free health care to all South Africans. All South Africans have the right to the necessary health services to promote, prevent, treat, rehabilitate, and alleviate illness. The quality of these health services is reliable and affordable for most people. The right to quality health services is determined by need, not socioeconomic status. National health insurance covers comprehensive health services, from community outreach and health promotion services to preventive services and other levels of medical services.

South Africa maintained an unemployment rate of 30% from 2000 to 2007, during which economic fluctuations grew more slowly. In the short-term causality test, different countries show different phenomena. The BRICS countries influence the global health agenda through diplomatic channels and growing economic power, and promote the transformation of the global health governance paradigm. Through technical cooperation, the BRICS countries strive to promote the capacity building of developing countries in the field of public health. The BRICS countries have increased the R&D and investment of related drugs, avoiding the passive situation caused by the high price of patent drugs and the backward medical technology. South Africa uses workforce employment as the dependent variable and health expenditure and economic fluctuation as the independent variable. The Fourier frequency is 0.1, which means that the relative changes of these three variables are relatively unsmooth and fast (the smaller the frequency number, the shorter the frequency interval). Due to the limitation of health expenditure data (2000-2019), there is no obvious delay in the three variables in Russia. Among them, Brazil and China have the highest two-level lagging data. In terms of medical policies, universal health coverage not only protects the health and wellbeing of individuals and communities, but also helps build a fair, stable, and united society. Achieving universal health coverage can provide employment and economic opportunities, and WHO estimates that out-of-pocket expenditures on health services put 100 million people into poverty each year.

## Conclusion and policy suggestions

We studied economic growth, the health expenditure, and the employment rate of the three variable of the Fourier Bootstrap ARDL model. We used the Fourier Bootstrap ARDL method, data from 2000 to 2019, to test whether correlations between economic fluctuations, health care expenditures, and employment rates among the BRICS countries were found. The results show that the Fourier Bootstrap ARDL analysis is useful for assessing the effects of different socio-economic scenarios in the context of different positions in health expenditure and economic policy in BRICS countries. In this empirical study, we find that there is no long-term cointegration relationship among the BRICS countries. Since there are only 20 years of annual data, the empirical data on time-series is relatively inadequate, but for the Fourier Bootstrap ARDL model, it has a loop detection function with lagging autoregression. Our research shows that economic fluctuations have no significant cointegration relationship between health expenditure and workforce employment rate. In turn, we used a reverse test to detect changes in workforce employment rate and economic fluctuation in health expenditure, and concluded that there was no cointegration relationship.

The basic goal of the health care system is to meet the health care needs of the people in the fairest and most efficient way, while ensuring the sustainability of government finances and the functioning of the health care system. Each country has its own context and reality environment, a unique medical system and a system that has developed over the years. Therefore, under different healthcare systems, countries will adopt effective policies that take into account the health needs of all citizens. Achieving equity, efficiency and sustainability is important from a healthcare system and policy perspective. This kind of sustainability refers to the sustainability of two aspects, one is the sustainability of government fiscal expenditures, and the other is the sustainability of the smooth operation of the medical system. The health system must run smoothly to take care of the health of all citizens. The most important key should be each country's health expenditure. Any expenditure that can promote national health should be broadly defined as health expenditure. In terms of policy, our suggestions for health spending and economic growth in the BRICS countries are as follows: 1 In the absence of a significant relationship between health expenditure and economic growth (China), the government should adopt a conservative and prudent health expenditure policy, targeting is to simply take care of people's health; 2. When health expenditure is an important factor in promoting economic growth (Brazil, India, Russia and South Africa), the government should aim to effectively provide health expenditure to promote economic growth and improve national health level; 3. If economic growth is an important factor driving the growth of health expenditure (Brazil, China and South Africa), the government does not need to pay more attention to health expenditure when the economic environment is not ideal policy. Proposing a governance direction that can significantly improve the economic situation and boost the economy is a top priority.

From a health perspective, health has two distinct effects on the economy. First, in terms of human resources, healthy citizens who are more productive and live longer reduce consumption of durable goods, but also increase savings rates; crowding out effect, reducing economic growth. But it will also drive the vigorous development of medical-related industries and promote economic growth. The government's policy is to guide the country toward development. If the BRICS countries find that the current health care system and system need to be coordinated in the process of development and transformation, they can take this opportunity to learn from the experience of other countries to improve, give full play to their own advantages and avoid repeating the mistakes, so as to achieve the coexistence and prosperity of medical and health expenditure and economic growth, and bring this experience to other countries in need of help in the world.

The Bootstrap ARDL simulation adapts to the bias of the narrow statistical environment used by McNown et al. (45). In particular, the Bootstrap ARDL test allows endogenous and feedback in the presence of variables. In addition, Pesaran et al. (58) degeneration cases # 1 or # 2 are only provided in the key-value ARDL Bounds test framework to test one of two possibilities. Yilanci et al. (57) allowed the use of Fourier frequency to smooth the breakpoints slightly. Therefore, empirical research using this method does not allow for two cases of degeneration at the same time, and it can be concluded that cointegration exists when it does not exist. Finally, the biggest limitation of this Fourier Bootstrap ARDL model is the variables. So far, the program from Yilanci et al. (57) and McNown et al. (45) can only use up to three variables. Therefore, there seems to be more causality in explanatory variables than in other models. If variables can be added to the model, we can increase the variables, which can strengthen the test of the model. In addition, our method using Fourier Bootstrap ARDL still has some related limitations; especially the data (source, missing, uniformity, etc.) may contain other related limitations, which may cause analysis bias. Of course, this may also be the direction in which this model can be improved in the future.

## Data availability statement

The original contributions presented in the study are included in the article/supplementary material, further inquiries can be directed to the corresponding authors.

## Author contributions

FL was responsible for organizing the progress of the paper, final revision, and finalization. M-CW was responsible for data collection and analysis, part of the manuscript writing, and submission. LG was responsible for guiding the topic selection, research methods, and comprehensive management of the structure and content of the article. All authors contributed to the article and approved the submitted version.

## Conflict of interest

The authors declare that the research was conducted in the absence of any commercial or financial relationships that could be construed as a potential conflict of interest.

## Publisher's note

All claims expressed in this article are solely those of the authors and do not necessarily represent those of their affiliated organizations, or those of the publisher, the editors and the reviewers. Any product that may be evaluated in this article, or claim that may be made by its manufacturer, is not guaranteed or endorsed by the publisher.

## Footnotes

^1^The authors compiled data information from https://databank.worldbank.org/source/world-development-indicators

^2^See: Global Spending on Health: A World in Transition, 2019, https://apps.who.int/iris/bitstream/handle/10665/~330357/WHO-HIS-HGF-HF-WorkingPaper-19.4-eng.pdf?ua=1

^3^IFC—a sister organization of the World Bank and member of the World Bank Group—is the largest global development institution focused on the private sector in developing countries.
https://www.ifc.org/wps/wcm/connect/corp_ext_content/ifc_external_corporate_site/home

^4^The authors calculated the BRICS data from the World Bank website. https://data.worldbank.org/

## References

[1] Marten R McIntyre D Travassos C Shishkin S Longde W Reddy S. An assessment of progress towards universal health coverage in Brazil, Russia, India, China, and South Africa (BRICS). Lancet. (2014) 384:2164–71. 10.1016/S0140-6736(14)60075-1 24793339 PMC7134989

[2] Rechel B Roberts B Richardson E Shishkin S Shkolnikov VM Leon DA. Health and health systems in the commonwealth of independent states. Lancet. (2013) 381:1145–55. 10.1016/S0140-6736(12)62084-4 23541055

[3] Harmer A. The BRICKS countries: a new force in global health. Bull World Health Organ. (2014) 92:394–5. 10.2471/BLT.14.030614 24940012 PMC4047813

[4] Grossman M. The Demand for Health: A Theoretical and Empirical Investigation. New York, NY: Columbia University Press (1972).

[5] Lucas R. On the mechanics of economic development. J Monet Econ. (1988) 22:3–42. 10.1016/0304-3932(88)90168-7

[6] Barro RJ. Health and economic growth. Ann Econ Finance. (1996) 14:329–66.

[7] McCoskey S Selden T. Health care expenditures and GDP: panel data unit root test results. J Health Econ. (1998) 17:369–76. 10.1016/S0167-6296(97)00040-4 10180923

[8] Kleiman E. The determinants of national outlay on health. In Perlman M, editor. The Economics of Health and Medical Care. London: Macmillan (1974). 10.1007/978-1-349-63660-0_5

[9] Newhouse JP. Medical care expenditures: a cross-national survey. J Hum Res. (1977) 12:115–25. 10.2307/145602 404354

[10] Leu RE. Public and private health services. In: Culyer AJ, Jonsson B, Editors. Complementarities and Conflicts. Oxford: Blackwell (1986). p. 41–63. Available online at: https://trove.nla.gov.au/version/22103532 (assessed May 12, 2022).

[11] Parkin D McGuire A Yule B. Aggregate health care expenditures and national income: is health care a luxury good? J Health Econ. (1987) 6:109–27. 10.1016/0167-6296(87)90002-6 10312163

[12] Gerdtham Ulf G Sogaard J Andersson F Jonsson B. An econometric analysis of health care expenditure: a cross-section study of the OECD countries. J Health Econ. (1992) 11:63–84. 10.1016/0167-6296(92)90025-V 10119757

[13] Gerdtham Ulf G Löthgren M. On stationarity and cointegration of international health expenditure and GDP. J Health Econ. (2000) 19:461–75. 10.1016/S0167-6296(99)00036-3 11010235

[14] Grigoli F Kapsoli J. Waste not, want not: the efficiency of health expenditure in emerging and developing economies. Rev Dev Econ. (2018) 22:384–403. 10.1111/rode.12346

[15] Ezziane Z. Essential drugs production in Brazil, Russia, India, China and South Africa (BRICS): opportunities and challenges. Int J Health Policy Manag. (2014) 3:365–70. 10.15171/ijhpm.2014.118 25489593 PMC4258887

[16] Jakovljevic M. Comparison of historical medical spending patterns among the BRICS and G7. J Med Econ. (2016) 19:70–6. 10.3111/13696998.2015.1093493 26366470

[17] Jakovljevic MB. BRIC's Growing Share of Global Health Spending and Their Diverging Pathways. Front Public Health. (2015) 3:135. 10.3389/fpubh.2015.00135 26000273 PMC4421927

[18] Jakovljevic M Potapchik E Popovich L Barik D Getzen TE. Evolving health expenditure landscape of the BRICS nations and projections to 2025. Health Econ. (2017) 26:844–52. 10.1002/hec.3406 27683202

[19] Tan SY Melendez-Torres GJ Pang T. Implementation of provider payment system reforms in the age of universal health coverage: a realist review of evidence from Asian developing countries. J Health Serv Res Policy. (2019) 24:279–87. 10.1177/1355819619842305 31007065

[20] Blomqvist A. Carter R. Is health care really a luxury? J Health Econ. (1997) 16:207–29. 10.1016/S0167-6296(96)00534-6 10169095

[21] Zaman K bin Abdullah A bin Khan A Nasir MRbM Hamzah TAAT. Dynamic linkages among energy consumption, environment, health and wealth in BRICS countries: Green growth key to sustainable development. Renew Sustain Energy Rev. (2016) 56:1263–71. 10.1016/j.rser.2015.12.010

[22] Stubbs T Kentikelenis A Stuckler D McKee M King L. The impact of IMF conditionality on government health expenditure: a cross-national analysis of 16 West African nations. Soc Sci Med. (2017) 174:220–7. 10.1016/j.socscimed.2016.12.016 28087084

[23] Yip WC Lee YC Tsai SL Chen B. Managing health expenditure inflation under a single-payer system: Taiwan's national health insurance. Soc Sci Med. (2019) 233:272–80. 10.1016/j.socscimed.2017.11.020 29548564

[24] Ghanbari A Basakha M. Investigating the effects of public health care expenditure on Iranian economic growth (1338-1383). Tahghighat E Eghtesadi. (2008) 43:187–224. Available online at: https://www.sid.ir/en/Journal/ViewPaper.aspx?ID=135305 (assessed March 21, 2022).

[25] Odior ES. Government expenditure on health, economic growth and long waves in A CGE micro-simulation analysis: the case of Nigeria. Eur J Econ Finance Adm Sci. (2011) 3:101–13. Available online at: https://ir.unilag.edu.ng/bitstream/handle/123456789/8182/19%20Government%20Expenditure%20on%20Health.pdf?sequence=1&isAllowed=y (assessed March 21, 2022).

[26] Piabuo SM Tieguhong JC. Health expenditure and economic growth - a review of the literature and an analysis between the economic community for central African states CEMAC and selected African countries. Health Econ Rev. (2017) 71:23. 10.1186/s13561-017-0159-1 28593509 PMC5462666

[27] Erçelik G. The relationship between health expenditure and economic growth in Turkey from 1980 to 2015. J Politic Econ Manage. (2018)1:1–8. Available online at: https://dergipark.org.tr/en/download/article-file/446368 (assessed May 12, 2022).

[28] Khoshnevis YS Khanalizadeh B. Air pollution, economic growth and health care expenditure. Econ Res. (2017) 30:1181–90. 10.1080/1331677X.2017.1314823

[29] Wang KM Lee YM Lin CL Tsai YC. The effects of health shocks on life insurance consumption, economic growth, and health expenditure: a dynamic time and space analysis. Sustain Cities Soc. (2018) 37:34–56. 10.1016/j.scs.2017.10.032

[30] Armanios DE Eesley CE Li J Eisenhardt KM. How entrepreneurs leverage institutional intermediaries in emerging economies to acquire public resources. Strateg Manag J. (2016) 38:1373–90. 10.1002/smj.2575

[31] Murthy NRV Ukpolo V. Aggregate health care expenditure in the United States: evidence from cointegration tests. Appl Econ. (1994) 26:797–802. 10.1080/00036849400000094

[32] Dickey DA Fuller WA. Distribution of the estimators for autoregressive time series with a unit root. J Am Stat Assoc. (1976) 74:427–31. 10.1080/01621459.1979.10482531

[33] Jakovljevic M Getzen TE. Growth of global health spending share in low and middle income countries. Front Pharmacol. (2016) 7:21. 10.3389/fphar.2016.00021 26903867 PMC4751681

[34] Jakovljevic MB Milovanovic O. Growing burden of non-communicable diseases in the emerging health markets: the case of BRICS. Front Public Health. (2015) 3:65. 10.3389/fpubh.2015.00065 25954740 PMC4407477

[35] Jakovljevic M Groot W Souliotis K. Editorial: health care financing and affordability in the emerging global markets. Front Public Health. (2016) 4:2. 10.3389/fpubh.2016.00002 26835444 PMC4720748

[36] Jakovljevic M Timofeyev Y Ekkert NV Fedorova JV Skvirskaya G Bolevich S. The impact of health expenditures on public health in BRICS nations. J Sport Health Sci. (2019) 8:516–9. 10.1016/j.jshs.2019.09.002 31720060 PMC6835015

[37] Yang X. Health expenditure, human capital, and economic growth: an empirical study of developing countries. Int J Health Econ Manage. (2020) 20:163–76. 10.1007/s10754-019-09275-w 31637560

[38] Hansen P King A. The determinants of health care expenditure: a cointegration approach. J Health Econ. (1996) 15:127–37. 10.1016/0167-6296(95)00017-8 10157426

[39] Jewell T Lee J Tieslau MA Strazicich M. Stationarity of health expenditures and GDP: Evidence from panel unit root tests with heterogeneous structural breaks. J Health Econ. (2003) 22:313–23. 10.1016/S0167-6296(02)00122-4 12606148

[40] Jakovljevic M Ogura S. Health economics at the crossroads of centuries - from the past to the future. Front Public Health. (2016) 4:115. 10.3389/fpubh.2016.00115 27376055 PMC4899886

[41] Zaidi S Saidi K. Environmental pollution, health expenditure and economic growth and in the Sub-Saharan Africa countries: panel ARDL approach. Sustain Cities Soc. (2018) 41:833–40. 10.1016/j.scs.2018.04.034

[42] Wang M Tao C. Research on the efficiency of local government health expenditure in china and its spatial spillover effect. Sustainability. (2019) 11:2469. 10.3390/su1109246931336960

[43] Haseeb M Kot S Hussain HI Jermsittiparsert K. Impact of economic growth, environmental pollution, and energy consumption on health expenditure and randd expenditure of ASEAN countries. Energies. (2019) 12:3598. 10.3390/en12193598

[44] Wang CM Hsueh HP Li F Wu CF. Bootstrap ARDL on health expenditure, CO[[sb]]2[[/s]] emissions, and GDP growth relationship for 18 OECD countries. Front Public Health. (2019) 7:324. 10.3389/fpubh.2019.00324 31824906 PMC6879421

[45] McNown R Sam CY Goh SK. Bootstrapping the autoregressive distributed lag test for cointegration. Appl Econ. (2018) 50:1509–21. 10.1080/00036846.2017.136664333838699

[46] Lee Y Kim SY. Public health law coverage in support of the health-related sustainable development goals (SDGs) among 33 Western Pacific countries. Global Health. (2019) 15:29. 10.1186/s12992-019-0472-z 30971269 PMC6458693

[47] Margerison-Zilko C Goldman-Mellor S Falconi A Downing J. Health impacts of the great recession: a critical review. Curr Epidemiol Rep. (2016) 3:81–91. 10.1007/s40471-016-0068-6 27239427 PMC4880023

[48] Hone T Mirelman AJ Rasella D Paes-Sousa R Barreto ML Rocha R. Effect of economic recession and impact of health and social protection expenditures on adult mortality: a longitudinal analysis of 5565 Brazilian municipalities. Lancet Global Health. (2019) 7:e1575–1583. 10.1016/S2214-109X(19)30409-7 31607469

[49] Bíróa A Elek P. Job loss, disability insurance and health expenditure. Lab Econ. (2020) 65:101856. 10.1016/j.labeco.2020.101856

[50] Raghupathi V Raghupathi W. Healthcare expenditure and economic performance: insights from the United States data. Front Public Health. (2020) 8:156. 10.3389/fpubh.2020.00156 32478027 PMC7237575

[51] Evans R Moore A Rees DM. The cyclical behaviour of the labour force participation rate in Australia. Australian Econ Rev. (2019) 52:94–106. 10.1111/1467-8462.12308

[52] Elshamy H. The relationship between unemployment and output in Egypt. Procedia Soc Behav Sci. (2013) 81:22–6. 10.1016/j.sbspro.2013.06.381

[53] Khrais I Al-Wadi M. Economic growth and unemployment relationship: an empirical study for MENA countries. Int J Manag Studies Res. (2016) 4:19–24. 10.20431/2349-0349.0412003

[54] Hjazeen H Seraj M Ozdeser H. The nexus between the economic growth and unemployment in Jordan. Future Bus J. (2021) 7:42. 10.1186/s43093-021-00088-3

[55] Gallant AR. On the bias in flexible functional forms and an essentially unbiased form. J Econom. (1981) 15:211–45.

[56] Gallant AR Souza G. On the asymptotic normality of fourier flexible form estimates. J Econom. (1991) 50:329–53. 10.1016/0304-4076(91)90024-8

[57] Yilanci V Bozoklu S Gorus MS. Are BRICS countries pollution havens? evidence from a bootstrap ARDL bounds testing approach with a fourier function. Sustain Cities Soc. (2020) 55:102035. 10.1016/j.scs.2020.102035

[58] Pesaran H Smith RJ Shin Y. Bound testing approaches to the analysis of level relationship. J Appl Econ. (2001) 16:289–326. 10.1002/jae.616

[59] Granger CWJ. Investigating causal relations by econometric models and cross-spectral methods. Econometrica. (1969) 37:424–38. 10.2307/1912791

[60] Perron P. The great crash, the oil price shock, and the unit root hypothesis. Econometrica. (1989) 57:1361–401. 10.2307/1913712

[61] Becker R Enders W Lee JA. Stationarity test in the presence of an unknown number of smooth breaks. J Time Ser Anal. (2006) 27:381–409. 10.1111/j.1467-9892.2006.00478.x

[62] Ludlow J Enders W. Estimating non-linear ARMA models using fourier coefficients. Int J Forecast. (2000) 16:333–47. 10.1016/S0169-2070(00)00048-0

[63] Christopoulos DK Leon-Ledesma MA. International output convergence, breaks, and asymmetric adjustment. Stud Nonlinear Dyn Econom. (2011) 15:1–33. 10.2202/1558-3708.1823

[64] Omay T. Fractional frequency flexible fourier form to approximate smooth breaks in unit root testing. Econ Lett. (2015) 134:123–6. 10.1016/j.econlet.2015.07.010

[65] Phillips PCB Perron P. Testing for a unit root in time series regression. Biometrika. (1988) 75:335–46. 10.1093/biomet/75.2.335

[66] Bai J Perron P. Estimating and testing linear models with multiple structural changes. Econometrica. (1998) 66:47–78. 10.2307/2998540

[67] Chan M, Brundtland, GH,. Universal Health Coverage: An Affordable Goal For All WHO Report (2016). Available online at: https://www.who.int/mediacentre/commentaries/2016/universal-health-coverage/en/ (accessed December 12, 2016).

[68] Ensor T. Informal payments for health care in transition economies. Soc Sci Med. (2004) 58:237–46. 10.1016/S0277-9536(03)00007-8 14604610

[69] Cheskin A March L. State–society relations in contemporary Russia: new forms of political and social contention. East European Politics. (2015) 31:261–73. 10.1080/21599165.2015.1063487

[70] Duggal R. Poverty & health: criticality of public financing. Indian J Med Res. (2007) 126:309–17. Available online at: https://media.proquest.com/media/hms/ORIG/1/IW9QE?_s=iOO8Ls2k8IxFF5JQxOAYE1ih7fw%3D (assessed May 12, 2022). 18032806

[71] Tafirenyika M. It's time to rethink medical insurance -A mobile phone plan in Kenya targets low-income groups Africa (2017).

